# Molecular Modification of Fluoroquinolone-Biodegrading Enzymes Based on Molecular Docking and Homology Modelling

**DOI:** 10.3390/ijerph16183407

**Published:** 2019-09-13

**Authors:** Si-cheng Liu, Shi-jun Sun, Peng Cui, Yi-fan Ding

**Affiliations:** School of Environment, Northeast Normal University, Changchun 130117, China; liusc413@nenu.edu.cn (S.-c.L.); cuip006@nenu.edu.cn (P.C.); dingyf671@nenu.edu.cn (Y.-f.D.)

**Keywords:** fluoroquinolone, degrading enzyme, homology modelling, molecular docking, density functional theory

## Abstract

To improve the biodegradation efficiency of fluoroquinolone antibiotics during sewage treatment, fluoroquinolone aerobic, anaerobic and facultative degrading enzymes for fluoroquinolone degradation were modified by molecular docking and homology modelling. First, amino acid residues of the binding sites of degrading enzymes for the target fluoroquinolones ciprofloxacin (CIP), norfloxacin (NOR) and ofloxacin (OFL) were analysed by the molecular docking method. The hydrophobic amino acid residues within 5 Å of the target fluoroquinolone molecules were selected as the modification sites. The hydrophobic amino acid residues at the modified sites were replaced by the hydrophilic amino acid residues, and 150 amino acid sequence modification schemes of the degrading enzymes were designed. Subsequently, a reconstruction scheme of the degrading enzyme amino acid sequence reconstruction scheme was submitted to the SWISS-MODEL server and a selected homology modelling method was used to build a new structure of the degrading enzyme. At the same time, the binding affinities between the novel degrading enzymes and the target fluoroquinolones (represented by the docking scoring function) were evaluated by the molecular docking method. It was found that the novel enzymes can simultaneously improve the binding affinities for the three target fluoroquinolones, and the degradation ability of the six modification schemes was increased by more than 50% at the same time. Among the novel enzymes, the affinity effect of the novel anaerobic enzyme (6-1) with CIP, NOR and OFL was significantly increased, with increases of 129.24%, 165.06% and 169.59%, respectively, followed by the facultative enzyme and aerobic enzyme. In addition, the designed degrading enzymes had certain selectivity for the degradation of the target quinolone. Among the novel enzymes, the binding affinities of the novel anaerobic enzyme (6-3) and CIP, the novel aerobic enzyme (3-6) and NOR, and the novel facultative enzyme (13-6) and OFL were increased by 149.71%, 178.57% and 297.12% respectively. Calculations using the Gaussian09 software revealed that the degradation reaction barrier of the novel degrading enzyme (7-1) and CIP NOR and OFL decreased by 37.65 kcal·mol^−1^, 6.28 kcal·mol^−1^ and 6.28 kcal·mol^−1^, respectively, which would result in efficient degradation of the target fluoroquinolone molecules. By analysing the binding affinity of the degrading enzymes before and after the modification with methanol, it was further speculated that the degradation effect of the modified aerobic degrading enzymes on organic matter was lower than that before the modification, and the increase or decrease in the degradation effect was less than 10%. The mechanism analysis found that the interaction between the modified amino acid residues of the degrading enzymes and the fluoroquinolone molecules increased. The average distance between the amino acid residues and the fluoroquinolone molecules represented a comprehensive affinity effect, and its value was positively correlated with the degradation effect of the novel degrading enzymes.

## 1. Introduction

Fluoroquinolone compounds are widely used as synthetic antimicrobial agents in the treatment of human and animal infectious diseases [1]. Their treatment after discharge to sewage treatment plants results in fluoroquinolone compound residues that are complex and difficult to remove, and they then flow into different environmental media; thus, they are considered to be environmentally harmful new environmental micropollutants [2,3].

Incomplete wastewater treatment can cause residual fluoroquinolone antibiotics to be discharged into the water environment through sewage treatment plants, resulting in certain bacterial resistance, so it is particularly important to improve the degradation of fluoroquinolones [4,5]. Antibiotics in sewage treatment plants are mainly eliminated by hydrolysis biodegradation and sludge adsorption [6], and the presence of microorganisms in the treatment process plays a key role in the removal of fluoroquinolones [7]. Fluoroquinolones exist in different treatment units such as aerobic anoxia and anaerobic, and each unit has different removal efficiencies [8]. The microbial degradation of fluoroquinolone antibiotics can be applied to the bioremediation process of sewage treatment plants to improve the removal efficiency of pollutants [9], and compared with other processes, there are abundant microorganisms in activated sludge, which is more suitable for enzymes modification.

At present, the research on the microbial degradation of fluoroquinolone antibiotics mainly focuses on the discovery of a single species of degrading microorganisms and the inference of degradation pathways. Martens et al. demonstrated the degradation of fluoroquinolone-enrofloxacin by wood rot fungi through experimental studies [10].

Based on the structure, function and catalytic mechanism, the rational design of the enzymes can identify the key amino acid residues related to the enzyme characteristics, and changes to the specific amino acid residues to modify the characteristics of an enzyme can be performed by means of molecular biology, such as substitutions, insertions or deletions [11]. Zhang et al. studied the catalytic activity, substrate specificity, mechanism and ligand binding affinity of cellulase Cel6A from *Thermobifida fusca*, and the results showed that the modified mutants increased the degradation activity of carboxymethyl cellulose [12]. Tadahiro et al. mutated Asn179 and Asp194 of endothermic alkaline cellulase to Lys, and found that the thermal stability of the enzymes was significantly improved. Enzymes designed to improve the microbial degradability of fluoroquinolones have rarely been used in previous studies [13].

In this paper, three common fluoroquinolone antibiotics—ciprofloxacin (CIP), norfloxacin (NOR) and ofloxacin (OFL)—were selected as the target molecules, and five kinds of aerobic, anaerobic and facultative bacteria were selected from the sewage treatment process. All the selected degradation bacteria can be found in sewage treatment plants. Although there was no specific study that indicates that they could degrade the target fluoroquinolone molecules, the results can be obtained by molecular docking of the selected degradation enzymes with the target fluoroquinolone molecules. First, the amino acid residues around the docking site of the degrading enzymes and the target fluoroquinolone molecules were determined by the molecular docking method. The structure of the novel degrading enzymes was constructed by a homology modelling method, and the binding affinity between the novel degrading enzymes and the target fluoroquinolone molecules was evaluated. In addition, the change in the degradation barrier before and after modification of the degrading enzyme modification was calculated with the Gaussian09 software (Gaussian Inc, Wallingford, CT, USA). The effects of degrading enzymes on the degradation of organic compounds before and after modification were evaluated, and the binding mechanism of novel degrading enzymes and target fluoroquinolones was analysed. It is expected that the future research on the efficient degradation and degradation mechanism of fluoroquinolone antibiotics will provide a theoretical basis for this research.

## 2. Materials and Methods

### 2.1. Molecular Docking Method

This study used the Tripos SYBYL-X 2.0 software (Tripos Inc., Saint Louis, MO, USA) for molecular docking. The selected target fluoroquinolone molecules, ciprofloxacin (CIP), norfloxacin (NOR), and ofloxacin (OFL) were constructed using the Sketch molecular module, taking the lowest energy conformation of ligand molecules as the dominant stable conformation. In the Tripos force field the molecular program Minimize was used to perform energy optimization. The Powell energy gradient method [14] was adopted, and the maximum number of iterations was 10,000, the convergence of the energy gradient was limited to 0.005 kJ/mol. The protease structure corresponding to the 15 commonly selected municipal wastewater treatment processes was derived from the Protein Data Bank. The active sites of each enzyme were determined by searching the National Center for Biotechnology Information (NCBI) for amino acid residues around the catalytic activity region of each enzyme. Before the molecular docking, the receptor protein obtained in the PDB protein library was pretreated. The treatment process included removing the ligand metal ions and water molecules, and adding polar hydrogen and point charges to expose the binding pocket [15]. In the docking process, the threshold value was set at 0.5 and the expansion coefficient was 0 (the default value). After the docking, the molecular morphology was generated according to the score of docking conformation score. The higher the score, the stronger the binding force is, and the conformation with the highest final score was selected as the optimal result of docking [16].

### 2.2. Homology Modelling Method

Homology modelling is a method for establishing a three-dimensional structure of a target protein that uses a similar protein and amino acid sequence of a known structure as a template [17]. In this paper, the NCBI database was used to query the amino acid residue sequences of the 15 selected enzymes. The key amino acid residues at the active sites of each enzyme were replaced by rational design and new amino acid sequences were designed. The amino acid sequence and the template enzymes of the new enzymes were respectively submitted to the SWISS-MODEL in the Automated Protein Modelling Server provided by the Glaxo Smith Kline centre (Geneva, Switzerland), and the homology modelling method was selected to obtain the molecular structure of the new enzymes [18]. A Ramachandran conformational map was generated by PROCHECK, which evaluates protein stereochemistry online, to evaluate each novel enzyme molecule structure constructed. The reliability of the model was considered to be satisfied when the Ramachandran conformational map showed that all of the amino acid residues of the protein were in the allowable region and the percentage of amino acid residues in the optimal region + allowable region + maximum allowable region was greater than 90% [19].

### 2.3. Gaussian Calculation Method

The Gaussian09 software (Gaussian Inc., Wallingford, CT, USA) [20] was used to optimize the molecular structure of the selected reactants CIP, NOR and OFL at the B3LYP/6-31G* basis set by density functional theory (DFT) [21]. The transition state (TS) and the reaction energy barrier (ΔE) were calculated at the same base group level, and complete the calculation of the simple harmonic frequency of each of the above substances was calculated. The transition state has one and only one virtual frequency, the intermediate has no virtual frequency, and the transition state was verified by the intrinsic reaction coordinate (IRC) approach.

## 3. Results and Discussion

### 3.1. Determination of Key Amino Acid Residues of Fluoroquinolone Degrading Enzymes Based on Molecular Docking Technology

**Figure 1:**
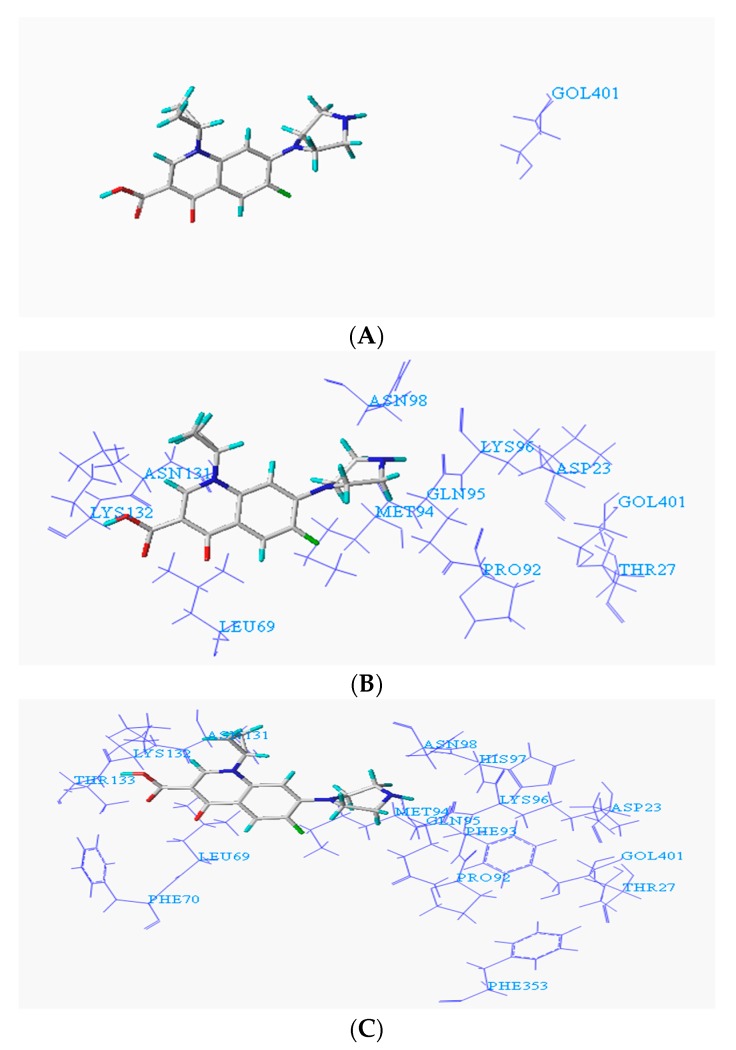
Conformation of ciprofloxacin (CIP) and degrading enzyme (PDB ID: 1YZP). (**A**) Within 1Å (GOL401); (**B**) Within 2Å (ASP23, THR27, LEU69, PRO92, GLN95, LYS96, ASN98, ASN131, LYS132, GOL401); (**C**) Within 5Å (ASP23, THR27, LEU69, PHE70, PRO92, MET94, GLN95, LYS96, HIS97, ASN98, ASN131, LYS132, THR133, PHE353).

To facilitate the determination of the binding sites of the degrading enzymes for the target fluoroquinolone molecules, the NCBI database was used to query the structural information of each enzyme (https://www.ncbi.nlm.nih.gov/protein/), and the amino acid residues in the catalytically active region of each enzyme were obtained. At the same time, the Surflex-Dock program in SYBYL-X 2.0 software was used to dock the degrading enzymes with the optimized target fluoroquinolone molecules. The program provides analysis of amino acid residues in three ranges 1 Å, 3 Å and 5 Å, respectively. Figure 1A–C show the conformation of the docked binding site of the CIP molecule and a degrading enzyme (PDB ID:1YZP) (for more information, the acid residues within 5 Å range were displayed, and the details given in Table 1 below).

**Figure 2:**
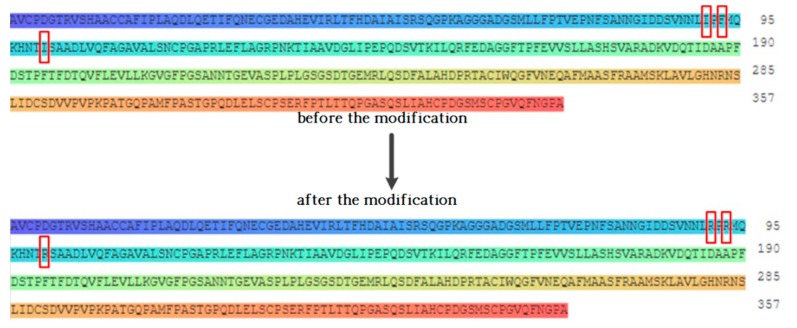
Schematic diagram of amino acid sequence changes before and after modification of degrading enzyme (PDB ID: 1YZP).

Since the amino acid residues within a certain distance between the binding site of the compound and the receptor were identified as key amino acid residues [22], the above amino acid residues were preliminarily identified as the key amino acid residues that directly interact with the target fluoroquinolone molecule. The hydrophobic effect of amino acids is considered to be the most important factor in the structure and stability of proteins, and the relative hydrophilicity/hydrophobicity of amino acids has a significant effect on protein-receptor binding and other intermolecular bio-cognitive processes [23]. Studies have shown that replacing hydrophilic amino acid residues that reside in binding sites for hydrophobic molecules with hydrophobic amino acid residues can improve the affinity between target molecules and degrading enzymes [24]. Therefore, by replacing the hydrophobic amino acid residues at the binding site of each degrading enzyme with hydrophilic amino acid residues, the affinity between the degrading enzymes and the target fluoroquinolone molecules can be increased, and the degradation ability of fluoroquinolones by the degrading enzymes can be further improved. Figure 2 shows the amino acid sequence changes before and after the modification of the degrading enzyme (PDB ID: 1YZP).

### 3.2. Modification of Fluoroquinolone Degrading Enzymes Based on Homology Modelling

#### 3.2.1. Design of the Modification Scheme for Fluoroquinolone Degrading Enzymes

Site-directed modification of degrading enzymes was performed by replacing single or multiple amino acids of the nine hydrophobic amino acid residues within the 5 Å range of the target fluoroquinolone molecule with hydrophilic amino acid residues. A total of 150 kinds of site-directed modification schemes of amino acid residues were designed. The novel degrading enzymes produced after the modification were molecularly docked with the target fluoroquinolone molecules, and finally 96 kinds of modification schemes with an affinity increase of more than 10% were screened out (Table 2).

#### 3.2.2. Homology Modelling and Model Validation of Fluoroquinolone Molecular Degrading Enzymes

**Figure 3:**
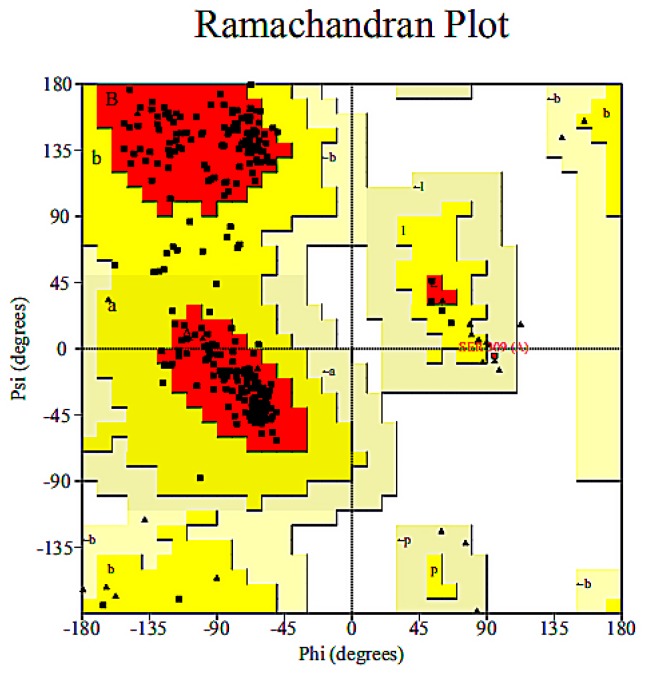
Ramachandran conformational map of a novel degrading enzyme (1-1).

To construct the three-dimensional molecular structure of the degrading enzymes after modification, the amino acid sequence of each degrading enzyme was obtained by the NCBI database. One or more hydrophobic amino acid residues were replaced with hydrophilic amino acid residues to obtain a new amino acid sequence, which was submitted to the SWISS-MODEL server. Enzymes with amino acid sequence similarity greater than 90% were used as template enzymes, and the homology modelling method was used to construct the three-dimensional molecular structures of the modified novel degrading enzymes. The homology of the novel degrading enzyme and template enzyme was more than 90%, which indicated that the selected template enzyme was reasonable [25]. A Ramachandran conformation map in PROCHECK online evaluation server was used to further verify and evaluate the structural rationality of the novel degrading enzyme constructed. The reliability of the model was considered to be satisfied when the Ramachandran conformational map showed that all of the amino acid residues of the protein were in the allowable region and the percentage of amino acid residues in the optimal region + allowable region + maximum allowable region was greater than 90% [19]. As shown in Figure 3, taking the novel degrading enzyme (1-1) as an example, it can be seen from the Ramachandran diagram shows that 89.9% of the amino acid residues of the constructed protein structure were in the optimal region, and 10.3% of the amino acid residues were in the allowable region, 0.2% is in the maximum allowable region. Only 0.2% of the amino acid residues were in the disallowed region, but these residues were not in the active region of the novel degrading enzyme (1-1). The above analysis indicates that the use of the homology modelling to construct three-dimensional structures of the novel degrading enzyme (1-1) is reasonable and reliable. The Ramachandran conformational maps of the 150 novel degrading enzymes were also analysed, and the results confirmed that the structure of the novel degrading enzyme protein structure satisfied the reasonability requirement, which states that the model of the amino acid residue percentage in the optimal region + allowable region + maximum allowable region should be greater than 90%.

### 3.3. Evaluation of the Binding Affinity between Novel Enzymes and Target Fluoroquinolones

To compare the changes in binding affinity between degrading enzymes and target fluoroquinolones before and after modification, the molecular docking of novel degrading enzymes and three target fluoroquinolones was completed by SYBYL-X 2.0 software (Tripos Inc., USA). The docking scoring function was calculated by considering the polar interaction, hydrophobic interaction, entropy, solvation and other factors between ligand and receptor. The higher the docking scoring function is, the stronger the binding affinity between ligand and receptor [16].

By comparing the docking function of each degrading enzyme before and after the modification with the target fluoroquinolone molecule, it was found that the score function of the novel degrading enzyme produced by replacing one or more hydrophobic amino acid residues at the binding site with hydrophilic amino acid residues at the binding site was increased or remained unchanged, and the binding affinity to CIP, NOR and OFL was increased simultaneously.

**Figure 4:**
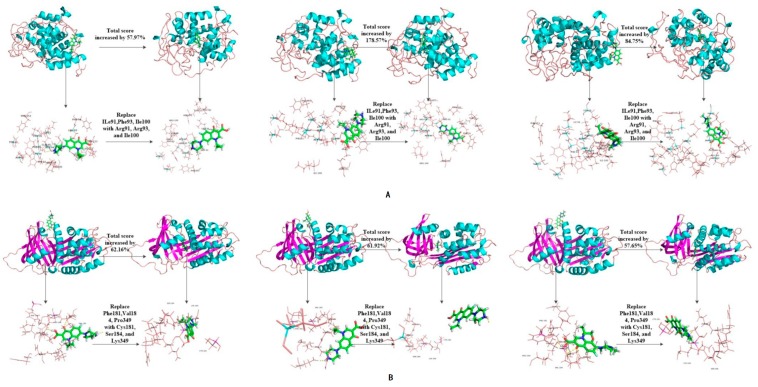

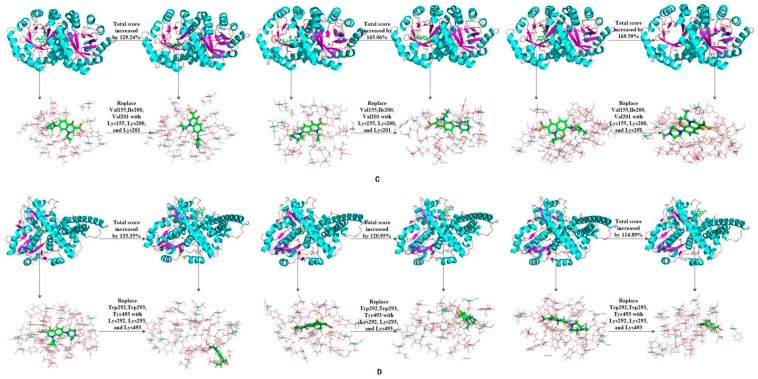

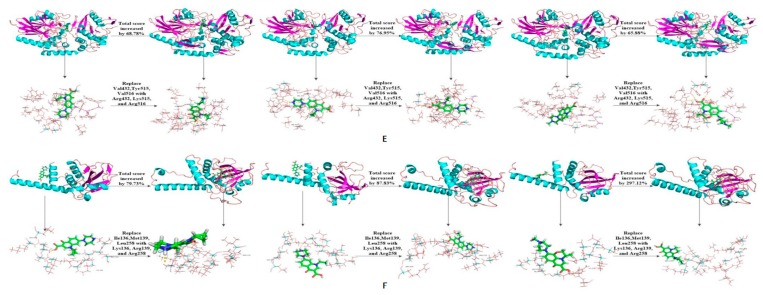
Schematic diagram of the structure of the target fluoroquinolone molecule before and after the degrading enzymes modification. (**A**–**F**) respectively represent the schematic diagram of the binding of the degrading enzymes with PDB ID of 1YZP, 4DTE, 3NQA, 1E1D, 1USH 2DYT and CIP, OFL, NOR).

The binding affinity of novel degrading enzymes and target fluoroquinolones increased by more than 10% in the 96 total modification schemes (Table 3), and among them, the binding affinities of six novel degrading enzymes and three target fluoroquinolone molecules increased by more than 50% simultaneously (bold numbers in Table 3). In summary, substituting a hydrophilic amino acid residue for a single hydrophobic residue or multiple hydrophobic amino acid residues at the binding active site can simultaneously increase the binding affinity between the degrading enzyme and the target fluoroquinolone molecules, thus, the degradation ability of the degrading enzymes was increased significantly. Among kinds of enzymes studied, the improved effect of the anaerobic degrading enzymes after modification were better than that of aerobic and facultative degrading enzymes. This may be due to the stronger binding affinity of anaerobic degrading enzyme to target forquinolone after modification. The binding structure of degrading enzymes and target fluoroquinolone molecules before and after modification is shown in Figure 4.

### 3.4. Calculation of the Energy Barriers for the Degradation of the Target Fluoroquinolone by Novel Degrading Enzymes

Huang et al. studied the pyrolysis process of lignin model compounds through density functional theory method, and calculated the reaction energy barrier and found that the lower the reaction energy barrier is, the better the pyrolysis reaction [26]. The catalytic degradation pathways of quinolones mainly include hydroxylation of c-8 and partial oxidation of piperazine groups [27,28]. To further explore the effect of the novel degrading enzymes on the molecular degradation of the target fluoroquinolone, the transition states (TS) and reaction energy barriers (ΔE) of the degradation reactions of the novel degrading enzymes and the target fluoroquinolone molecules that were simultaneously increased by 50% were calculated by Gaussian09 software at the B3LYP/6-31G* basis level. The calculation formula of the reaction energy barrier is shown in Equation (1), and the calculation results are shown in Table 4.

ΔE = E (TS) − ΣE (Reactant)(1)

Compared with the template enzyme, the degradation energy barriers of the novel enzyme 7-1 and CIP, NOR and OFL were reduced, as determined by calculating the reaction energy barrier of the novel enzyme catalysed molecular degradation reaction of the fluoroquinolones. The degradation barrier of other novel degradation enzymes and different target fluoroquinolones also decreased to different degrees, as shown in the Table 5. In summary, the selected degrading enzyme modification scheme can reduce the reaction energy barrier of the target fluoroquinolone molecular degradation reaction, indicating that the target fluoroquinolone molecule is more susceptible to degradation reactions with the novel degrading enzyme.

### 3.5. Analysis of the Effect of the Degrading Enzymes before and after Modification on the Degradation of Organic Matter

Methanol is a common carbon source in urban sewage treatment plants [29]. To further investigate whether the degradation effect of the degrading enzymes on organic matter was affected after the modification, the binding affinity (scoring function) of the degrading enzymes in Table 4 and methanol was analysed by the molecular docking method (Table 6).

Table 6 shows that the binding affinity of the degrading enzyme and methanol will changes after modification. The binding affinity of the novel aerobic degrading enzymes with methanol was increased or decreased by no more than 10%, and the anaerobic and facultative degradation bacteria had relatively large changes. Therefore, it was speculated that the modified aerobic degrading enzymes had little impact on the degradation of organic matter.

### 3.6. Mechanism Analysis of the Binding of the Novel Degrading Enzymes and Target Fluoroquinolones

**Figure 5:**
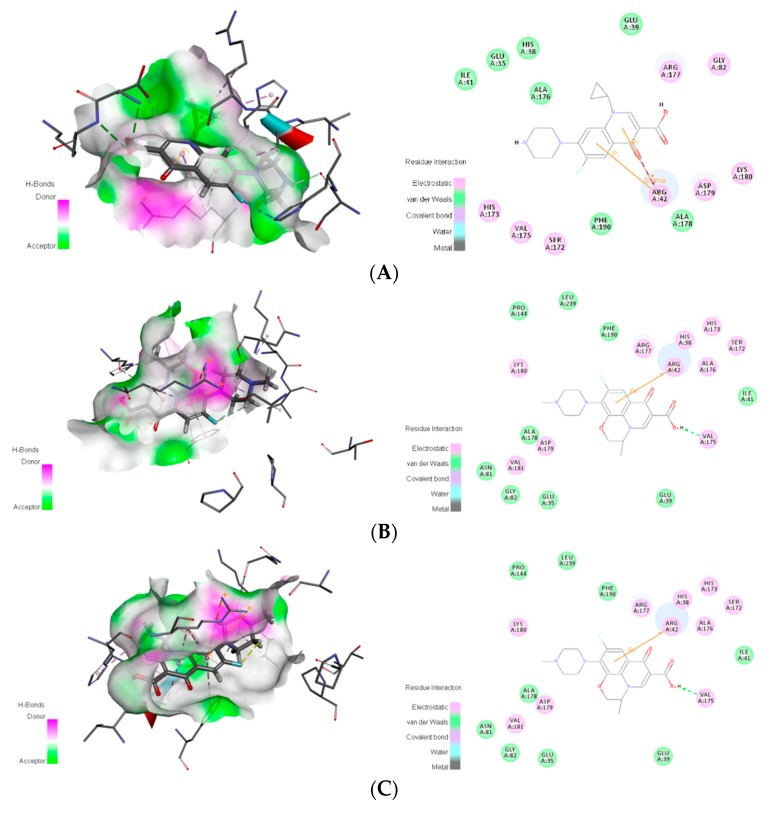
Schematic diagram of the binding for a novel degrading enzyme (3-6) and a target fluoroquinolone molecule. The left is the schematic diagram of the hydrogen donor, and the right is the 2D model diagram: (**A**) ciprofloxacin; (**B**) norfloxacin; (**C**) ofloxacin.

To further analyse the binding of the novel degrading enzymes and the target fluoroquinolone molecules, the interaction between the degrading enzymes in Table 4 and the target fluoroquinolone molecules was analysed by Discovery Studio 4.0 software (BIOVIA Inc, Shenzhen, China). The types of interactions between the target fluoroquinolone molecules and the amino acids surrounding the degrading enzyme are listed in Table 7. A 2D diagram of the interaction between novel degrading enzyme (3-6) and the target fluoroquinolone molecules as an example is shown in Figure 5.

The interaction analysis between novel degrading enzymes (3-6) and the target fluoroquinolones showed that when the novel degrading enzyme (3-6) was combined with CIP, Glu35, His38, Glu39, Ile41, Ala176, and Ala178 have van der Waals force, and had electrostatic interactions with Arg42, Gly82, Ser172, His173, Val175, Arg177, Asp179, Lys180, and His38; Ser172, Val175, Asp179 generated carbon-hydrogen bond interactions, generated halogen interactions with His173, had conventional hydrogen bonding with Asp179 and Lys180, generated π-Alkyl interactions with His38, Arg42, Ala176, and generated alkyl interactions with Arg177; and Arg42 produced a π interactions pair and π-sigma effect. When the novel degrading enzyme (3-6) was combined with NOR, Glu35, Glu39, Ile41, Gly82, Ala176, and Phe190 had van der Waals forces, and had electrostatic interactions with His38, Arg42, Ser172, His173, Val175, Arg177, Ala178, Asp179, and His38; Val175 and Asp179 generated carbon-hydrogen bond interactions, generated halogen interactions with His173, produced π-alkyl interactions with Arg42 and Ala176, and generated π interactions and π-cation with Arg42. When the novel degrading enzyme (3-6) was combined with OFL, residues Glu35, Glu39, Ile41, Asn81, Gly82, Pro144, Ala178, Phe190, and Leu239 had van der Waals forces, electrostatic interactions occurred with His38, Arg42, Ser172, His173, Val175, Arg177, Asp179, Lys180 and Val181; generated carbon-hydrogen bonds were formed with His38 and Arg177; generated halogen interactions were formed with His173; conventional hydrogen bonding formed with Val175; π-alkyl interactions were formed with His38, Arg42 and Ala176. The alkyl interaction interacted with Ala178, Lys180 and Val181, generated a π-interacting pair and π-cation interactions with Arg42, generated π-sigma interactions with Ala176 and generated π-π T-shaped interactions with Phe190.The binding of the compound and the receptor protein can be made stable by forming multiple forces with the surrounding amino acids [30]. Table 7 shows that the binding of the degrading enzyme and the target fluoroquinolone molecules. The force of the template enzyme and the novel degrading enzyme after docking with the target fluoroquinolone molecule were compared and analysed. The results showed that compared with the template-degrading enzyme, the number of action types formed by the novel degrading enzyme (3-6) binding to the CIP molecule was increased. Among the novel degrading enzymes that bind to OFL, compared with the template degrading enzymes, the type of force generated by the combination of the novel degrading enzyme (6-1) and the OFL molecule was increased. It may make the binding of the novel degrading enzymes and target fluoroquinolones more stable. And the comparison showed that van der Waals force and electrostatic force were always present before and after modification of degradation enzyme. It can be speculated that the two forces may be the main force, which may be more stable than other forces. To further explore the mechanism of binding between the degrading enzyme and the target fluoroquinolone molecule before and after the modification, the distances between the amino acid residues and the target fluoroquinolone molecules before and after the substitution of amino acid residues in the modification scheme with a 50% increase of in binding affinity were measured respectively, and the mean values were calculated (Table 8).

As shown in Table 8, when 1GYC was used as a template, the average distance between amino acid residues Ile91, Phe93, Ile100 and CIP, NOR and OFL was 8.10 Å, 8.57 Å and 7.03 Å, respectively. According to the modification scheme, the amino acid residues Ile91, Phe93 and Ile100 were replaced with Arg91, Arg93 and Arg100, respectively, and the mean distances between the three new amino acid residues and the CIP NOR OFL molecules was 19.80 Å (>8.10 Å), 24.17 Å (>8.57 Å) and 24.20 Å (>7.03 Å), respectively. Similarly, the remaining schemes in Table 6 were analysed, and the mean distance between the replaced amino acid residue and the target fluoroquinolone molecules was increased compared with the previous replacement. The docking scoring function of the novel degrading enzyme and the target fluoroquinolone molecule produced by replacing the amino acid residues in Table 3 was higher than the template enzyme. It can be inferred that the greater the average distance between the replaced amino acid residues and the target fluoroquinolone molecule, the greater the binding force between them is [31]. It can be further concluded that the average distance between the replaced amino acid residues and the target fluoroquinolone molecules is positively correlated with the degradation effect of the novel enzyme on the target fluoroquinolone molecule.

## 4. Conclusions

In this paper, molecular docking and homology modelling methods were used to modify the aerobic anaerobic and facultative enzymes for fluoroquinolone degradation, and six novel degrading enzyme modification schemes with an increase in binding affinity of more than 50% to the target fluoroquinolone molecules were selected. Among them, the anaerobic novel degrading enzyme (6-1) had the most significant effect, and the binding affinity with CIP, NOR and OFL was increased by more than 120%, 129.24%, 165.06% and 169.59%, respectively. The improvement in the degradation capacity of the anaerobic degrading enzymes after modification was better than that of the aerobic and facultative degrading enzymes. In addition, the novel degrading enzymes had a certain selectivity for molecular degradation of the target fluoroquinolones. In all the modification schemes, the binding affinities of novel anaerobic enzyme (6-3) and CIP, novel aerobic enzyme (3-6) and NOR, and novel facultative enzyme (13-6) and OFL were increased by 149.71%, 178.57% and 297.12% respectively. After modification, the degrading enzyme significantly improved the degradation ability of the target fluoroquinolone molecule, but the degradation effect of the degrading enzyme on organic matter may change after modification. Through the analysis of the binding affinities of the degrading enzymes and methanol before and after modification, it was further speculated that the degradation effect of modified aerobic degradation bacteria on organic matter was small compared with that before the modification, and the increase or decrease range was not more than 10%. Based on the above discussion, it can be concluded that the degrading enzymes in aerobic processes may be suitable for modification to obtain a novel degrading enzyme that can improve the degradation effect of fluoroquinolone and that has little impact on the degradation effect of organic matter. The above conclusions can provide theoretical guidance for future studies on the efficient degradation mechanism of fluoroquinolones and the modification of degrading enzymes in urban sewage treatment plants.

## Figures and Tables

**Table 1:** Statistics of amino acid residues within 5 Å range of the target fluoroquinolone molecule.

Degrading Bacteria Type	PDB ID	Bacterium	Amino Acid Residues within 5 Å Range of the Target Fluoroquinolone Molecule		
			Combined with CIP	Combined with NOR	Combined with OFL
Aerobic bacteria	1ARP	*Arthromyces ramosus*	Ile81, Ala82, His83, Ser84, Arg104, Ile108, His110, Val112, Ser113, Phe144, Asn143	Ile81, Ala82, His83, Ser84, Asn85, Ile86, Glu101, Arg104, Ile108, Phe114	Ile81, Ala82, His83, Ser84, Asn85, Ile86, Leu88, Thr97, Ile100, Glu101, Arg104
	1EI5	*Ochrobactrum anthropi*	Asn155, Asp481, Cys61, Ser62, Ala482, Ala290, Leu509, Tyr153, Pro483, Ser152, Arg295, Cys296, His287, Tyr151, Trp508, Thr375, Glu409, Ser376, Ser379, Phe373, Pro377, Arg406, Val392, Glu378	Cys296, Gln297, Arg295, Ala290, His287, Ala318, Glu315, Asn275, Ser319, Tyr270, Tyr153, Pro483, Tyr151, Leu509, Thr375, Glu409, Trp508, Ser376, Arg406	Trp299, Gln297, Lys65, His287, Glu278, Leu276, Ser62, His277, Asn275, Ser319, Tyr270, Ala290, Pro269, Asn106, Tyr153, Ser408, Tyr151, Val392, Trp508
	1GYC	*Trametes versicolor*	Asn340, Asn341, Ala329, Phe338, Ser343, Asn333, Asn336, Thr335, Met57, Thr56, Asn54, Leu158, Ala155, Ala24, Tyr152, Thr154, Asp23, His153, Arg22, Phe20, Pro319, Ala325, Leu326, Glu381, Asn327, Thr383, Thr438, Asn436, Pro385, Asp435, Ala386, Ile203, Arg121, Ala1, Ser33, Thr144, His216, Val30, Leu174, Pro123, Val145, Pro34, Asn249, Ala186, Ile298, Leu35, Asp234, Thr147, Phe31, Ile188, Val187, Leu232, Val297, Ile146, Pro32, Asn172, Leu185, Thr219, Asn217, Glu142, Ser143, Asn251, Gln252	Pro319, Leu326, Thr383, Asn340, Pro385, Ile298, Thr219, Asn217, Asn249, Asn251, Gln252, Ala325, Thr438, Ala329, Asp435, Leu232, Val297, Asp234, His216, Glu142, Ser143, Glu381, Asn327, Asn436, Asn341, Ala386, Pro123, Val145, Ser33, Pro32, Asn172, Phe31, Asn189, Leu174, Phe338, Ser343, Thr335, Arg121, Thr147, Leu35, Pro34, Ile171, Ile188, Phe31, Val30, Ala186, Asn333, Asn336, Met57, Thr56, Tyr152, Asn54, His153, Asp23, Phe20, Leu158, Ala155, Thr154, Ala24, Arg22	Pro319, Asn436, Pro385, Asn340, Asn341, Thr335, Gln252, Val145, Pro123, Val187, Pro32, Leu174, Glu381, Ala325, Thr383, Asp435, Ala386, Phe338, Asn333, Asn249, Thr219, Leu232, Ile188, His216, Asp234, Ile298, Thr438, Leu326, Asn327, Ala329, Ser343, Asn336, Asn251, Thr144, Asn189, Pro34, Asn217, Ser33, Asn172, Ala24, Met57, Arg22, Tyr152, Thr56, His153, Glu142, Ser143, Leu35, Ile188, Ile146, Thr147, Val297, Asn54, Asp23, Phe20, Ala155, Thr154, Leu158
	1YZP	*Phanerochaete chrysosporium*	Phe70, Thr133, Leu69, Lys132, Phe353, Asn131, Pro92, Met94, Gln95, Phe93, Lys96, Asn98, Thr27, His97, Asp23	Val73, Thr72, Pro356, Pro71, Phe70, Leu69, Leu68, Phe353, Ile91, Asn131, Pro92, Met94, Gln95, Phe93, Lys96, Thr27, Asp23	Leu69, Phe353, Ala102, Asn131, Pro130, Arg129, Pro92, Met94, Ser101, Asp104, Phe93, Ile100, Asn98, Lys96, Thr27, His97, Thr99, Asp23
	4DTE	*Nocardia*	Phe181, Pro371, Glu373, Gln370, Pro349, Thr183, Thr369, Asn26, Val184, Asn185, Arg25, Asp24	Ser55, Glu373, Gln370, Arg25, Asn185, Asp24, Thr183, Val184	Glu373, Gln370, Asn26, Arg25, Asp24, Asn185, Thr369, Thr183, Phe181, Val184, Pro349
Anaerobic bacteria	3NQA	*Methanothermobacter thermautotrophicus*	Pro46, Asp20, Asp70, Lys42, Tyr206, Arg203, Ala18, Lys72, Ile96, Leu123, Val20, Ile200, Met126, His128, Pro180, Ala184, Val155, Ser127, Gln125, Ser179, Val182, Pro157, Ser158	Pro46, Tyr45, Asp20, Lys72, Asp70, Ile96, Lys42, Tyr206, Ala18, Arg203, Val201, Ser204, Ile200, Met126, Val155, Ser127, Pro180, Ala184, Gln185, Val182	Pro46, Asp20, Asp70, Lys72, Ile96, Lys42, Tyr206, Ala18, Arg203, Leu123, Val201, Ile200, Thr124, Met126, Val155, His128, Pro180, Ala184, Glu125, Ser127, Ser179, Gln185, Val182, Pro157, Ser158
	1E1D	*Desulfovibrio vulgaris*	Ala435, Tyr437, Cys459, Cys406, Ala497, Asp407, Cys434, Tyr493, Lys496, Glu494, Tyr161, Ser291, Glu268, Met269, Trp292, Trp293, Thr71, Ile70, His244, Gln294, Asn287, Tyr288, His266, Cys312, Asn311, Ser242, Gln295, Asn311, Cys312, Thr310, Glu298, Phe299, Leu308, Leu313, Val314	Tyr161, Thr310, Leu308, Asn311, His244, Lys496, Cys312, Ser242, Glu494, Met296, Ala497, Cys459, Glu268, His266, Phe299, Tyr493, Trp292, Gln295, Cys406, Cys434, Trp293, Asp407, Ser291, Ala435	Ile70, Tyr61, Lys496, Glu494, Thr310, Ala497, Asn311, Tyr493, Cys312, Leu313, Cys459, His244, Leu308, Cys406, Glu268, Ser242, Asp407, Cys434, Ala435, Trp292, Trp293, His266, Ser291, Gln294, Tyr437, Phe299, Glu298
	3EZX	*Methanosarcina barkeri*	Met48, Lys49, Met72, Val46, Leu44, Ser45, His106, Ile108, Ile40, Leu155, Pro187, Ala186, His110, Leu112, Glu203, Asn204, Val113, Ala205, Ala202, Ala206, Ala208	Met48, His110, Leu155, Ala154, Ser153, Val113, Ala186, Asn204, Val151, Val188, Glu203, Phe183, Ala208, Ala202, Met182, Thr201	Ser37, Ile105, Met66, Asp107, Leu62, His106, Ile108, Ile65, His110, Leu155, Met48, Val51, Lys49, Ala186, Asp53
	5D5P	*Methanococcus maripaludis*	Leu87, Lys119, Met116, Asn84, Leu83, Arg121, Ile122, Leu83, Met80	Lys119, Leu87, Leu83, Ile122, Met80, Arg79	Arg79, Thr78, Leu83, Asn84, Leu81
	1BFM	*Methanothermus fervidus*	Phe67, Lys68, Val64, Lys69, Ile39, Asp38, Lys68, Met35, Glu34, Ile31	Val64, Ile39, Leu32, Asp38, Phe67, Lys68, Lys69, Glu34, Met35	Val64, Ile39, Lys68, Lys69, Phe67, Asp38, Met35, Glu34
Facultative bacteria	1USH	*Escherichia coli*	Val32, Asn497, His43, Trp291, Thr518, Glu290, His117, Asp84, Asp41, Asn517, Gln254, Asp255, Val516, Asn116, His252, Ser253, Ile178, Tyr515, His217, Asn229, Tyr221	Val432, Asn497, Phe520, His43, Asp510, Thr518, Asn517, Gln254, Asp255, Lys265, Val516, Tyr515, His117, Asp84, Ser253, His252, Ile178, Asn116, Asn229, Tyr221, His220	Ile521, His43, Val432, His117, Trp291, Thr518, Asp84, Lys293, Asn497, Asn116, Glu290, Ile178, Asn517, Val516, Gln254, His252, His289, Tyr515, Ser253, Asp255
	1BLI	*Bacillus licheniformis*	Phe190, Met197, Leu196, Tyr193, His105, Glu189, His235, Ser334, Tyr56, Lys234, Ala232, Leu335, Cal233, Trp263, Asp231, Asp328, Tyr262	Val102, Met197, Tyr56, Leu196, Leu230, Asp231, Arg229, Val233, Ala232, Lys234, Glu261, Trp13, Tyr262, Trp263, His327, Asp328, Ser334, Gln333, Leu335	Tyr56, Leu196, His235, Asp231, Arg229, Val233, Ala232, Tyr193, Lys234, Phe190, Glu261, Trp13, Glu189, Tyr262, His327, Trp263, Asp328, Ser334, Gln333, Ser264, Leu335
	2DYT	*Saccharomyces cerevisiae*	Val250, Gln252, Arg251, Asp133, Lys256, Arg254, Glu134, Ile136, Arg255, Asp279, Gln137, Leu258, Glu140, Ile141	Val250, Asp133, Arg251, Ile136, Glu134, Arg254, Arg255, Gln137, Met139, Asp279, Asp138, Glu140, Ile141, Lys142	Asp133, Glu134, Arg251, Arg254, Glu257, Gln137, Asp138, Arg255, Leu258, Ser280, Asp279
	4GW3	*Proteus mirabilis*	Phe143, Thr138, Ile139, Tyr24, Ile140, Phe16, Ile255, Met219, Ala135, Gis254, Phe136, Leu222, Leu13, His11, His78, Leu234, Ala153, Ser79, Leu44, Ala46, Gln80, Ala156, Phe47, Val105, Leu157, Leu160, Agr53	Asp149, Ser144, Ile19, Phe143, Ile140, Asp152, Val20, Ile139, Phe136, Ala153, Phe16, Leu13, Phe47, Ala156, Tyr24, Leu157, Ile255, His254, Leu234, Ser79	His146, Asp149, Ser144, Phe143, Ile140, Ile139, Phe136, Asp152, Ile19, Val20, Ala153, Phe16, Ile255, Tyr24, Leu13, Ala156, Phe47, Leu157, Leu234, His254, Ala46, Ser79, Leu160, Gln80
	1EFP	*Paracoccus denitrificans*	Ala155, Asp153, Ala154, Phe152, Lys21, Ser151, Asp17, Thr149, Lys272, Glu251, Ala150, Arg194, Pro250, Ser192, Ala249, Ala193, Thr191, Val248	Asp153, Ala154, Phe152, Lys21, Ser151, Asp17, Thr149, Lys272, Glu251, Ala150, Arg194, Pro250, Ser192, Ala249, Ala193, Thr191, Val248	Asp270, Ser271, Lys272, Val196, Val248, Ala249, Glu251, Pro250, Ser151, Ala150, Leu190, Thr191, Arg194, Ala193, Thr149, Glu189, Phe152, Ser192, Asp153, Lys21, Ala154

Note: The PDB ID is the Protein Data Bank identification.

**Table 2:** Modification of degrading enzymes with affinity increase of more than 10%.

Degrading Bacteria Type	PDB ID	Novel Degrading Enzyme Number	Hydrophobic Amino Acid Residues (Before Modification)	Hydrophilic Amino Acid Residues (After Modification)
Aerobic bacteria	1GYC	1-1	Ala325	Asp
		1-2	Pro385	Glu
		1-3	Pro34	His
			Leu232	Lys
		1-4	Pro123	Glu
			Leu232	Glu
			Val297	His
		1-5	Val187	Cys
			Ile298	Gln
			Val297	Asp
		1-6	Val187	Gln
			Ile298	Gln
			Val297	Gln
		1-7	Pro32	Ser
			Tyr152	Thr
			Ala386	Lys
	1ARP	2-1	Ile100	Cys
			Phe114	Asn
		2-2	Ile86	Cys
			Val112	His
		2-3	Leu88	Asn
			Ile108	Thr
		2-4	Leu88	Gln
			Ile108	Cys
		2-5	Leu88	Ser
			Ile108	His
		2-6	Leu88	Lys
			Ile108	Asn
		2-7	Leu88	Arg
			Ile108	Asp
		2-8	Leu88	Cys
			Ile108	Thr
		2-9	Leu88	Cys
			Phe114	Thr
			Ile100	Thr
	1YZP	3-1	Ile91	Lys
			Phe93	Lys
			Ile100	Lys
		3-2	Ile91	Asp
			Phe93	Asp
			Ile100	Asp
		3-3	Ile91	Glu
			Phe93	Glu
			Ile100	Glu
		3-4	Phe70	Lys
			Ile91	Lys
			Ile100	Lys
		3-5	Phe70	Glu
			Ile91	Glu
			Ile100	Glu
		3-6	Ile91	Arg
			Phe93	Arg
			Ile100	Arg
		3-7	Ile91	Asp
			Phe93	Asp
			Ile100	Asp
		3-8	Phe70	Arg
			Ile91	Arg
			Ile100	Arg
		3-9	Phe70	Asp
			Ile91	Asp
			Ile100	Asp
	1EI5	4-1	Ala290	Lys
			Phe373	Lys
			Val392	Lys
		4-2	Ala290	Asn
			Phe373	Asn
			Val392	Asn
		4-3	Ala290	Lys
			Phe373	Lys
		4-4	Ala290	Asp
			Phe373	Asn
	4DTE	5-1	Phe181	Cys
			Val184	Cys
			Pro349	His
		5-2	Phe181	Cys
			Val184	Ser
			Pro349	Lys
Anaerobic bacteria	3NQA	6-1	Val155	Lys
			Ile200	Lys
			Val201	Lys
		6-2	Val155	Asp
			Ile200	Asp
			Val201	Asp
		6-3	Val155	Asp
			Ile200	Lys
			Val201	Lys
		6-4	Val155	Asp
			Ile200	Asp
			Val201	Lys
		6-5	Val155	Asp
			Ile200	Asp
			Val201	Arg
		6-6	Val155	Arg
			Ile200	Lys
			Val201	Asp
		6-7	Val155	Asn
			Ile200	Asn
			Val201	Lys
		6-8	Val155	Asn
			Ile200	Asp
			Val201	Lys
		6-9	Val155	Asp
			Ile200	Asn
			Val201	Lys
		6-10	Val182	Lys
			Ile200	Lys
			Val201	Lys
	1E1D	7-1	Trp292	Lys
			Trp293	Lys
			Tyr493	Lys
		7-2	Trp292	Arg
			Trp293	Lys
			Tyr493	Lys
		7-3	Trp292	Arg
			Trp293	Lys
			Tyr493	Arg
		7-4	Trp292	Arg
			Trp293	Arg
			Tyr493	Arg
		7-5	Trp292	Arg
			Trp293	Arg
			Tyr493	Lys
		7-6	Trp292	Arg
			Trp293	Asp
			Tyr493	Lys
		7-7	Trp292	Asp
			Trp293	Asp
			Tyr493	Lys
		7-8	Trp292	Asp
			Trp293	Asp
			Tyr493	Asp
		7-9	Trp292	Lys
			Trp293	Asp
			Tyr493	Asp
		7-10	Trp292	Lys
			Trp293	Lys
			Tyr493	Asp
	3EZX	8-1	Leu155	Arg
			Ala186	Lys
			Ala202	Asp
		8-2	Leu155	Asn
			Ala186	Lys
			Ala202	Asp
		8-3	Leu155	Asn
			Ala186	Lys
			Ala202	Asn
		8-4	Leu155	Arg
			Ala186	Lys
			Ala202	Asn
	5D5P	9-1	Met80	Arg
			Leu83	Arg
			Leu87	Arg
		9-2	Met80	Asp
			Leu83	Lys
			Leu87	Lys
		9-3	Met80	Arg
			Leu83	Lys
			Leu87	Glu
		9-4	Met80	Thr
			Leu83	Arg
			Leu87	Arg
	1BFM	10-1	Met35	Glu
			Ile39	Gln
			Phe67	Glu
Facultative bacteria	1USH	11-1	Val432	Arg
			Tyr515	Arg
			Val516	Arg
		11-2	Val432	Lys
			Tyr515	Lys
			Val516	Lys
		11-3	Val432	Arg
			Tyr515	Lys
			Val516	Lys
		11-4	Val432	Arg
			Tyr515	Arg
			Val516	Lys
		11-5	Val432	Arg
			Tyr515	Lys
			Val516	Arg
		11-6	Val432	Lys
			Tyr515	Lys
			Val516	Arg
		11-7	Val432	Lys
			Tyr515	Arg
			Val516	Lys
		11-8	Val432	Lys
			Tyr515	Arg
			Val516	Arg
		11-9	Val432	Asp
			Tyr515	Asp
			Val516	Asp
		11-10	Val432	Arg
			Tyr515	Asp
			Val516	Asp
	1BLI	12-1	Leu196	Arg
			Ala232	Asp
			Val233	Arg
	2DYT	13-1	Ile136	Arg
			Met139	Arg
			Leu258	Arg
		13-2	Ile136	Glu
			Met139	Glu
			Leu258	Glu
		13-3	Ile136	Lys
			Met139	Lys
			Leu258	Lys
		13-4	Ile136	Arg
			Met139	Arg
			Leu258	Lys
		13-5	Ile136	Arg
			Met139	Lys
			Leu258	Lys
		13-6	Ile136	Lys
			Met139	Arg
			Leu258	Arg
		13-7	Ile136	Glu
			Met139	Arg
			Leu258	Arg
		13-8	Ile136	Glu
			Met139	Glu
			Leu258	Arg
		13-9	Ile136	Arg
			Met139	Glu
			Leu258	Lys
	4GW3	14-1	Phe136	Arg
			Ile139	Arg
			Ile140	Arg
		14-2	Phe136	Lys
			Ile139	Lys
			Ile140	Lys
		14-3	Phe136	Arg
			Ile139	Lys
			Ile140	Lys
		14-4	Phe136	Arg
			Ile139	Lys
			Ile140	Arg
		14-5	Phe136	Lys
			Ile139	Lys
			Ile140	Arg
		14-6	Phe136	Asp
			Ile139	Lys
			Ile140	Arg
		14-7	Phe136	Arg
			Ile139	Arg
			Ile140	Asp
		14-8	Phe136	Arg
			Ile139	Asp
			Ile140	Arg
	1EFP	15-1	Ala150	Arg
			Phe152	Arg
			Pro250	Arg
		15-2	Ala150	Lys
			Phe152	Lys
			Pro250	Lys
		15-3	Ala150	Arg
			Phe152	Lys
			Pro250	Lys
		15-4	Ala150	Arg
			Phe152	Arg
			Pro250	Lys
		15-5	Ala150	Asp
			Phe152	Arg
			Pro250	Arg
		15-6	Ala150	Asp
			Phe152	Asp
			Pro250	Arg
		15-7	Ala150	Asp
			Phe152	Asp
			Pro250	Asp
		15-8	Ala150	Arg
			Phe152	Lys
			Pro250	Asp

**Table 3:** Docking scoring function of novel degrading enzymes and target fluoroquinolone molecules.

Degrading Bacteria Type	PDB ID	Novel Degrading Enzyme	With the CIP			With the NOR			With the OFL		
			Pre-Modification Scoring Function	Modified Scoring Function	Change Rate (%)	Pre-Modification Scoring Function	Modified Scoring Function	Change Rate (%)	Pre-Modification Scoring Function	Modified Scoring Function	Change Rate (%)
Aerobic bacteria	1GYC	1-1	3.27	3.97	21.41	2.13	4.48	110.33	2.20	2.43	10.45
		1-2		4.31	31.80		2.80	31.46		3.44	56.36
		1-3		4.49	37.31		2.79	30.99		3.32	50.91
		1-4		4.35	33.03		2.58	21.13		3.07	39.55
		1-5		4.00	22.32		4.38	105.63		3.83	74.09
		1-6		3.60	10.09		3.84	80.28		3.76	70.91
		1-7		4.13	26.30		2.88	35.21		2.70	22.73
	1ARP	2-1	3.36	5.30	57.74	3.01	5.36	78.07	3.84	4.78	24.48
		2-2		6.16	83.33		7.90	162.46		4.95	28.91
		2-3		6.37	89.58		7.18	138.54		5.15	34.11
		2-4		6.29	87.20		7.05	134.22		5.07	32.03
		2-5		6.12	82.14		8.15	170.76		5.45	41.93
		2-6		7.05	109.82		7.74	157.14		5.66	47.40
		2-7		6.37	89.58		7.13	136.88		6.06	57.81
		2-8		6.07	80.65		7.49	148.84		5.11	33.07
		2-9		6.26	86.31		6.39	112.29		6.38	66.15
	1YZP	3-1	3.64	5.94	63.19	2.66	7.29	174.06	2.95	5.87	98.98
		3-2		5.85	60.71		6.40	140.60		6.59	123.39
		3-3		6.86	88.46		5.64	112.03		6.54	121.69
		3-4		5.81	59.62		7.31	174.81		5.74	94.58
		3-5		5.77	58.52		6.09	128.95		6.62	124.41
		**3-6**		**5.75**	**57.97**		**7.41**	**178.57**		**5.45**	**84.75**
		3-7		5.85	60.71		6.40	140.60		6.59	123.39
		3-8		5.91	62.36		7.40	178.20		5.77	95.59
		3-9		6.01	65.11						
	1EI5	4-1	5.08	6.43	26.57	3.74	6.07	62.30	4.44	5.55	25.00
		4-2		5.77	13.58		5.23	39.84		5.16	16.22
		4-3		5.67	11.61		4.74	26.74		4.90	10.36
		4-4		5.60	10.24		4.71	25.94		4.93	11.04
	4DTE	5-1	3.70	4.07	10.00	3.23	3.72	15.17	3.66	4.21	15.03
		**5-2**		**6.00**	**62.16**		**5.23**	**61.92**		**5.77**	**57.65**
Anaerobic bacteria	3NQA	6-1	1.71	3.92	129.24	2.69	7.13	165.06	−3.42	2.38	169.59
		6-2		2.71	58.48		3.46	28.62		−0.35	89.77
		6-3		4.27	149.71		3.76	39.78		0.09	102.63
		6-4		2.42	41.52		4.00	48.70		−2.62	23.39
		6-5		2.03	18.71		4.55	69.14		−2.19	35.96
		6-6		2.39	39.77		5.09	89.22		−1.16	66.08
		6-7		3.94	130.41		3.74	39.03		−1.34	60.82
		6-8		4.24	147.95		3.73	38.66		−2.86	16.37
		6-9		3.12	82.46		5.46	102.97		2.75	180.41
		6-10		3.65	113.45		4.84	79.93		2.68	178.36
	1E1D	**7-1**	−18.08	**6.03**	**133.35**		**4.11**	**120.95**	−28.47	**4.24**	**114.89**
		7-2		5.47	130.25		3.13	115.95		4.86	117.07
		7-3		5.27	129.15		4.01	120.44		3.12	110.96
		7-4		5.18	128.65		3.86	119.67		4.23	114.86
		7-5		5.09	128.15		3.61	118.40		4.39	115.42
		7-6		4.62	125.55		3.04	115.49		4.33	115.21
		7-7		5.61	131.03		2.85	114.53		4.23	114.86
		7-8		5.28	129.20		3.90	119.88		4.30	115.10
		7-9		5.03	127.82		2.84	114.48		4.18	114.68
		7-10		5.25	129.04		3.68	118.76		2.97	110.43
	3EZX	8-1	4.09	6.63	62.10	4.00	4.51	12.75	4.60	5.24	13.91
		8-2		6.62	61.86		5.93	48.25		5.97	29.78
		8-3		4.62	12.96		6.33	58.25		5.34	16.09
		8-4		4.56	11.49		4.91	22.75		5.39	17.17
	5D5P	9-1	4.58	5.11	11.57	2.74	4.28	56.20	4.17	5.22	25.18
		9-2		6.92	51.09		3.10	13.14		5.47	31.18
		9-3		6.02	31.44		4.28	56.20		5.78	38.61
		9-4		5.04	10.04		5.23	90.88		4.90	17.51
	1BFM	10-1	3.12	4.02	28.85	3.46	4.38	26.59	3.05	4.29	40.66
Facultative bacteria	1USH	11-1	3.78	5.97	57.94	3.08	4.53	47.08	3.37	5.21	54.60
		11-2		5.57	47.35		4.01	30.19		4.45	32.05
		11-3		5.65	49.47		3.87	25.65		4.35	29.08
		11-4		6.11	61.64		4.27	38.64		3.72	10.39
		**11-5**		**6.38**	**68.78**		**5.45**	**76.95**		**5.59**	**65.88**
		11-6		5.89	55.82		5.62	82.47		5.42	60.83
		11-7		5.89	55.82		4.46	44.81		5.25	55.79
		11-8		5.63	48.94		4.88	58.44		5.30	57.27
		11-9		4.40	16.40		5.24	70.13		4.77	41.54
		11-10		4.74	25.40		6.02	95.45		5.26	56.08
	1BLI	12-1	3.51	5.65	60.97	3.49	4.42	26.65	4.26	4.73	11.03
	2DYT	13-1	2.96	3.53	19.26	2.63	4.01	52.47	1.39	3.03	117.99
		13-2		4.71	59.12		3.58	36.12		3.71	166.91
		13-3		5.95	101.01		3.37	28.14		4.49	223.02
		13-4		6.77	128.72		4.34	65.02		4.17	200.00
		13-5		4.30	45.27		3.74	42.21		3.92	182.01
		**13-6**		**5.32**	**79.73**		**4.94**	**87.83**		**5.52**	**297.12**
		13-7		4.80	62.16		4.00	52.09		3.11	123.74
		13-8		4.49	51.69		4.81	82.89		3.37	142.45
		13-9		4.69	58.45		3.00	14.07		3.14	125.90
	4GW3	14-1	3.60	4.70	30.56	2.61	5.56	113.03	3.66	5.36	46.45
		14-2		4.36	21.11		3.93	50.57		5.22	42.62
		14-3		4.53	25.83		4.77	82.76		6.41	75.14
		14-4		4.27	18.61		4.82	84.67		6.05	65.30
		14-5		4.00	11.11		4.54	73.95		5.09	39.07
		14-6		5.54	53.89		5.21	99.62		4.99	36.34
		14-7		5.28	46.67		4.87	86.59		5.27	43.99
		14-8		5.30	47.22		4.55	74.33		5.04	37.70
	1EFP	15-1	4.48	5.59	24.78	3.12	4.36	39.74	3.32	5.06	52.41
		15-2		5.89	31.47		3.70	18.59		5.22	57.23
		15-3		5.62	25.45		5.36	71.79		3.81	14.76
		15-4		5.63	25.67		4.16	33.33		4.76	43.37
		15-5		5.70	27.23		4.61	47.76		4.94	48.80
		15-6		5.30	18.30		4.00	28.21		5.24	57.83
		15-7		4.93	10.04		4.73	51.60		4.77	43.67
		15-8		5.26	17.41		4.87	56.09		4.95	49.10

Note: The bold type in the table is the new enzyme with 50% improved binding affinity.

**Table 4:** Energy barrier calculation of degradation reaction between novel enzymes and target fluoroquinolones.

Molecular Target	Enzyme	E_reactant_/(a.u.)	E_TS_/(a.u.)	ΔE/(a.u.)	ΔE/(kcal·mol^−1^)
CIP	1YZP	−1148.37	−1224.11	−75.74	−47,527.61
	3-6	−1148.37	−1224.11	−75.74	−47,527.61
	4DTE	−1148.37	−1224.13	−75.75	−47,533.88
	5-2	−1148.37	−1224.05	−75.68	−47,489.96
	3NQA	−1148.37	−1224.12	−75.75	−47,533.88
	6-1	−1148.37	−1223.93	−75.56	−47,414.66
	1E1D	−1148.37	−1224.03	−75.66	−47,477.41
	7-1	−1148.37	−1224.09	−75.72	−47,515.06
	1USH	−1148.37	−1224.11	−75.74	−47,527.61
	11-5	−1148.37	−1224.11	−75.74	−47,527.61
	2DYT	−1148.37	−1223.98	−75.61	−47,446.03
	13-6	−1148.37	−1224.11	−75.74	−47,527.61
NOR	1YZP	−1110.31	−1186.01	−75.70	−47,502.51
	3-6	−1110.31	−1186.02	−75.71	−47,508.78
	4DTE	−1110.31	−1186.04	−75.73	−47,521.33
	5-2	−1110.31	−1185.98	−75.67	−47,483.68
	3NQA	−1110.31	−1185.96	−75.65	−47,471.13
	6-1	−1110.31	−1185.87	−75.56	−47,414.66
	1E1D	−1110.31	−1185.96	−75.65	−47,471.13
	7-1	−1110.31	−1186.03	−75.72	−47,515.06
	1USH	−1110.31	−1185.85	−75.53	−47,395.83
	11-5	−1110.31	−1186.05	−75.74	−47,527.61
	2DYT	−1110.31	−1186.04	−75.73	−47,521.33
	13-6	−1110.31	−1186.04	−75.73	−47,521.33
OFL	1YZP	−1262.93	−1338.58	−75.65	−47,471.13
	3-6	−1262.93	−1338.60	−75.67	−47,483.68
	4DTE	−1262.93	−1338.67	−75.74	−47,527.61
	5-2	−1262.93	−1338.67	−75.74	−47,527.61
	3NQA	−1262.93	−1224.12	38.81	24,353.66
	6-1	−1262.93	−1338.66	−75.73	−47,521.33
	1E1D	−1262.93	−1338.66	−75.73	−47,521.33
	7-1	−1262.93	−1338.67	−75.74	−47,527.61
	1USH	−1262.93	−1338.58	−75.65	−47,471.13
	11-5	−1262.93	−1338.67	−75.74	−47,527.61
	2DYT	−1262.93	−1338.67	−75.74	−47,527.61
	13-6	−1262.93	−1338.55	−75.62	−47,452.31

**Table 5:** Degradation barriers between novel degradation enzymes and different target fluoroquinolones.

Target Fluoroquinolone Molecules	Novel Degradation Enzyme Number	Degree of Reduction (%)
CIP	7-1	6.60
	13-6	6.60
NOR	3-6	6.82
	7-1	6.82
	11-5	6.82
OFL	3-6	5.99
	7-1	6.00
	11-5	6.00

**Table 6:** Dating function and variation range of degrading enzyme and methanol before and after transformation.

PDB ID	Scoring Function	Novel Degrading Enzyme Number	Scoring Function	Amplitude of Variation
1YZP	2.09	3-6	2.07	−0.96%
4DTE	2.06	5-2	2.21	7.28%
3NQA	2.40	6-1	3.01	25.42%
1E1D	2.38	7-1	2.67	12.1%
1USH	2.36	11-5	3.77	59.75%
2DYT	2.57	13-6	2.13	−17.12%

**Table 7:** Interaction between novel degrading enzymes and target fluoroquinolones.

Target Molecular	Degrading Enzyme	Interaction Type	Amino Acid Residue
CIP	1YZP	Van der Waals	Glu143, Pro144, Gln145, Lys180, Thr219, Gly220
		Electrostatic	Arg42, Ser78, Asn81, Val181
		Carbon hydrogen bond interaction	Ser78
		Conventional hydrogen bond interaction	Arg42
		Alkyl interaction	Pro144
		π-Alkyl interaction	Val181
		Amide-π stacked interaction	Lys180, Val181
	3-6	Van der Waals	Glu35, His38, Glu39, Ile41, Ala176, Ala178
		Electrostatic	Arg42, Gly82, Ser172, His173, Val175, Arg177, Asp179, Lys180
		Carbon hydrogen bond interaction	His38, Ser172, Val175, Asp179
		Conventional hydrogen bond interaction	Asp179, Lys180
		Halogen (fluorine) interaction	His173
		Alkyl interaction	Arg177
		π-Alkyl interaction	His38, Arg42, Ala176
		π Interaction pair	Arg42
		π-Sigma interaction	Arg42
	4DTE	Van der W#aals	Pro219, Gly360, Ile225, Ser224, His356
		Electrostatic	Ser226, Gln355, Arg255, Glu223, Glu217, Lys357, His252
		Carbon hydrogen bond interaction	Glu217, Gly360
		Conventional hydrogen bond interaction	Ser226, Glu223
		Halogen (fluorine) interaction	Ser226, Gln355
		Alkyl interaction	Arg255
		π-Alkyl interaction	Lys357
		π-Cation interaction	Arg255
	5-2	Van der Waals	Phe15, Leu27, Ala28, Ser30, Pro31, Val83, Leu161, Phe163, Ile225, Val316, Lys317, Leu318, Phe351
		Electrostatic	Leu29, Phe352, Ile354
		Carbon hydrogen bond interaction	Leu27, Pro31
		Alkyl interaction	Ala28, Leu161, Leu318
		π-Donor hydrogen bond interaction	Ile354
		π-Alkyl interaction	Leu161
	3NQA	Van der Waals	Val151, Asn111, Ala113
		Electrostatic	Asn153, Leu149, Leu110, Gly150, Arg107, Val120, Lys152, Glu119, Glu114
		Carbon hydrogen bond interaction	Val120, Gly150, Leu149
		Halogen (fluorine) interaction	Leu110
		π-Alkyl interaction	Leu110
		π-Cation interaction	Arg107
		π-Donor hydrogen bond interaction	Arg107
	6-1	Van der Waals	Leu49, Ser50, Gly52, Ala86, Ala90
		Electrostatic	Val48, Leu49, Gly52, Met53, Asp54, Lys89
		Carbon hydrogen bond interaction	Val48, Leu49
		Alkyl interaction	Met53, Lys89, Ala90
		π-Alkyl interaction	Ala90
	1E1D	Van der Waals	Asp483, Ile481, Val509, Val486, Met549
		Electrostatic	Asp480, Asn482, Asn535, Lys537, Lys510, Gly511, Arg513, Lys553, Ser487, Tyr488, Arg399
		Carbon hydrogen bond interaction	Asp480, Asn482
		Conventional hydrogen bond interaction	Asp480, Lys537, Arg513
		Halogen (fluorine) interaction	Asn535
		π-Alkyl interaction	Tyr488
	7-1	Van der Waals	Lys293, Lys411, Ser414, Tyr439, Leu442
		Electrostatic	Ser291, Gln294, Asp407, Gly408, Arg409, Thr417, Tyr437, Arg438
		Carbon hydrogen bond interaction	Ser291, Asp407, Gly408
		Halogen (fluorine) interaction	Gly294, Asp407
		π-Interaction pair	Arg438
		π-Cation interaction	Arg438
	1USH	Van der Waals	Tyr221, Glu225, Asn511, Lys512, Pro513, Tyr515, Val516
		Electrostatic	His226, Gly227, Ser228, Asn229, Asp510, Gly514, Asn517
		Carbon hydrogen bond interaction	His226, Ser228, Asn229, Lys512, Tyr515
		Coventional hydrogen bond interaction	His226, Ser228
		Halogen (fluorine) interaction	Asp510
		π-Alkyl interaction	Tyr515
		π-π T-shaped interaction	Tyr515
		π-Lone pair interaction	Asp510
	11-5	Van der Waals	His220, Tyr221, Ser253, Asp510, Asn517
		Electrostatic	Asp222, Glu225, His226, Gly227, Ser228, Asn229, Asn255, Lys265, Pro513, Gly514, Lys515, Arg516
		Carbon hydrogen bond interaction	His220, Asp255, Lys515
		Coventional hydrogen bond interaction	Ser228, Asp255, Gly514, Lys515
		Halogen (fluorine) interaction	Glu225
		Alkyl interaction	Lys515
		π-π Stacked interaction	Tyr221
	2DYT	Van der Waals	Ile129, Ile132, Leu135, Leu243, Val289, Phe293, Ser296, Thr306
		Electrostatic	Ile288, Lys292, His303
		Conventional hydrogen bond interaction	Ile288
		Halogen (fluorine) interaction	His303
		Alkyl interaction	Ile132
		π-Alkyl interaction	Lys292
		π-Sigma interaction	Thr295
		Amide-π stacked interaction	Thr295, Ser296
	13-6	Van der Waals	Val86, Glu87, Pro89, Asp90, Val91, Glu152, Phe153, Ala155, Gly158, Leu159
		Electrostatic	Lys78, Gly88, Asn154, Pro299, Ser300, Ile301, Gln302, His303
		Carbon hydrogen bond interaction	Asn154, Gln302
		Conventional hydrogen bond interaction	Asn154, His303
		Alkyl interaction	Val86, Pro89
		π-π T-shaped interaction	His303
NOR	1YZP	Van der Waals	His46, Ile141, Pro144, Gln145, Asn218, Gly220, Leu239
		Electrostatic	Arg42, Ser78, Ala79, Asn80, Asn81, Pro142, Glu143, Lys180, Val181, Gln183, Thr219
		Carbon hydrogen bond interaction	Ala79, Thr219
		Conventional hydrogen bond interaction	Arg42, Asn81
		Halogen (fluorine) interaction	Arg42, Ser78, Ala79, Asn81
		Alkyl interaction	Ala79, Pro144
		π-Sigma interaction	Val181
		π-Interaction pair	Arg42
	3-6	Van der Waals	Glu35, Glu39, Ile41, Gly82, Ala176, Phe190
		Electrostatic	His38, Arg42, Ser172, His173, Val175, Arg177, Ala178, Asp179
		Carbon hydrogen bond interaction	His38, Val175, Asp179
		Halogen (fluorine) interaction	His173
		π-Alkyl interaction	Arg42, Ala176
		π-Interaction pair	Arg42
		π-Cation interaction	Arg42
	4DTE	Van der Waals	Pro219, Gly360, His356, Ser224, Tyr220, Ile225
		Electrostatic	Glu223, Glu217, Ser226, Arg255, Lys357, His252, Gln355
		Carbon hydrogen bond interaction	Glu223, Glu217, Gly360
		Conventional hydrogen bond interaction	Glu223, Ser226
		Halogen (fluorine) interaction	Gln355
		Alkyl interaction	Arg255
		π-Alkyl interaction	Lys357
		π-Cation interaction	Arg255
		π interaction pair	Arg255
	5-2	Van der Waals	Phe15, Ala28, Ser30, Pro31, Val83, Leu161, Phe163, Val316, Lys317, Leu318, Leu353
		Electrostatic	Leu27, Leu29, His162, Phe352, Ile354
		Carbon hydrogen bond interaction	Leu29, Pro31, Phe352
		Alkyl interaction	Leu161, Val316, Leu318
		π-Sigma interaction	Leu161, Ile354
		π-Donor hydrogen bond interaction	Ile354
	3NQA	Van der Waals	Ala113, Gly150, Val151, Leu149
		Electrostatic	Lys152, Glu119, Leu110, Asn111, Val120, Glu114, Arg107, Asn153
		Carbon hydrogen bond interaction	Glu119, Gly150
		Conventional hydrogen bond interaction	Glu114
		Halogen (fluorine) interaction	Leu110
		π-Cation interaction	Arg107
		π-Sigma interaction	Leu110
		π-Anion interaction	Glu114
		π-Interaction pair	Arg107
	6-1	Van der Waals	Ser50, Ser52
		Electrostatic	Val48, Leu49, Gly52, Met53, Asp54
		Carbon hydrogen bond interaction	Val48
		Halogen (fluorine) interaction	Val48, Leu49
		π-Donor hydrogen bond interaction	Met53
		π-Anion interaction	Asp54
	1E1D	Van der Waals	Asp483, Val509, Val486, Ile512, Met549
		Electrostatic	Asp480, Lys510, Asn535, Lys553, Lys537, Gly511, Arg513, Asn482, Tyr488, Ser487
		Carbon hydrogen bond interaction	Asp480, Asn482
		Coventional hydrogen bond interaction	Asp480, Lys537, Arg513
		π-Alkyl interaction	Tyr488
	7-1	Van der waals	Lys293, Lys411, Ser414, Leu442
		Electrostatic	Ser291, Gln294, Asp407, Gly408, Arg409, Thr417, Tyr437, Arg438
		Carbon hydrogen bond interaction	Ser291, Asp407, Gly408, Arg409
		Halogen (fluorine) interaction	Gln294, Asp407
		π-Cation interaction	Arg438
	1USH	Van der Waals	Gly227, Pro513
		Electrostatic	Ser228, Asn229, Ser253, Asp510, Asn511, Lys512, Gly514, Tyr515, Asn517
		Carbon hydrogen bond interaction	Asp510, Val516
		Conventional hydrogen bond interaction	Ser228, Asn517
		Halogen (fluorine) interaction	Ser228, Lys512
		π-π T-shaped interaction	Tyr515
	11-5	Van der Waals	Gly227, Val434, Thr501, Asn511, Lys512
		Electrostatic	Ser228, Asn229, Asp510, Pro513, Gly514, Lys515, Arg516, Asn517
		Carbon hydrogen bond interaction	Asp510
		Coventional hydrogen bond interaction	Ser228, Arg516, Asn517
		Halogen (fluorine) interaction	Asn517
		π-Alkyl interaction	Lys515
	2DYT	Van der Waals	Ile129, Asp131, Ile132, Leu135, Ile136, Leu243, Val289, Ser291, Phe293, Ser296, Thr306
		Electrostatic	Ile288, Lys292, Thr295, His303
		Carbon hydrogen bond interaction	Ile288
		Halogen (fluorine) interaction	His303
		Alkyl interaction	Ile132, Leu243, Lys292
		π-Alkyl interaction	Lys292
		Amide-π stacked interaction	Thr295, Ser296
	13-6	Van der Waals	Val86, Glu87, Gly88, Pro89, Asp90, Ala155, Lys156, Gly158
		Electrostatic	Asp77, Lys78, Asn154, Gly157, Leu159, Ser300, Gln302, His303
		Carbon hydrogen bond interaction	Asn154
		Alkyl interaction	Val86, Pro89
		π-Alkyl interaction	Val86
		π-Anion interaction	Asp77
OFL	1YZP	Van der Waals	His46, Ile141, Pro144, Val181, Thr219
		Electrostatic	Arg42, Ala79, Asn81, Pro142, Glu143, Glu145, Lys180, Gln183
		Carbon hydrogen bond interaction	Ala79, Pro142, Glu143, Gln145
		Coventional hydrogen bond interaction	Asn81
		Halogen (fluorine) interaction	Asn81
		Alkyl interaction	Ala79, Pro144
	3-6	Van der Waals	Glu35, Glu39, Ile41, Asn81, Gly82, Pro144, Ala178, Phe190, Leu239
		Electrostatic	His38, Arg42, Ser172, His173, Val175, Arg177, Asp179, Lys180, Val181
		Carbon hydrogen bond interaction	His38, Arg177
		Conventional hydrogen bond interaction	Val175
		Alkyl interaction	Ala178, Lys180, Val181
		π-Alkyl interaction	His38, Arg42, Ala176
		π-Interaction pair	Arg42
		π-Cation interaction	Arg42
		π-Sigma interaction	Ala176
		π-π T-shaped interaction	Phe190
	4DTE	Van der Waals	Asn201, Tyr220, Pro219, His356
		Electrostatic	Arg255, Glu223, Glu217, Lys357, Ser226, Glu355, Ser24, Ile225
		Carbon hydrogen bond interaction	Glu223
		Conventional hydrogen bond interaction	Lys357
		Alkyl interaction	Pro219
		π-Anion interaction	Glu217
	5-2	Van der Waals	Phe15, Ala28, Ser30, Pro31, Leu77, Glu80, Val83, Leu161, Phe163, Ile225, Val316, Leu318, His356
		Electrostatic	Leu27, Leu29, Tyr32, Phe352, Ile354
		Carbon hydrogen bond interaction	Ser30
		Conventional hydrogen bond interaction	Phe352
		Halogen (fluorine) interaction	Ile354
		Alkyl interaction	Ala28, Leu161, Val316, Leu318, Ile354
		π-Alkyl interaction	Ala28, Pro31, Ile354
	3NQA	Van der Waals	Arg118, Glu119, Val120, Glu114, Arg107, Leu149
		Electrostatic	Glu119, Arg118, Ala113
		Carbon hydrogen bond interaction	Leu110
		Alkyl interaction	Leu110
		π-Alkyl interaction	Arg118, Glu119, Val120, Glu114, Arg107, Leu149
	6-1	Van der Waals	Val48, Leu49, Ser50, Lys89, Ala90
		Electrostatic	Val48, Leu49, Ser50, Gly52, Met53, Asp54
		Carbon hydrogen bond interaction	Val48, Leu49, Ser50
		Alkyl interaction	Val48, Leu49, Met53
		π-Donor hydrogen bond interaction	Met53
	1E1D	Van der Waals	Asp480, Asp483, Val509, Val486, Met549
		Electrostatic	Lys510, Asn482, Ser487, Arg399, Asn535, Lys537, Gly511, Arg513, Lys553, Tyr488
		Carbon hydrogen bond interaction	Asp480, Asn482, Lys510
		Conventional hydrogen bond interaction	Lys537, Arg513
		Halogen (fluorine) interaction	Asn535
		Alkyl interaction	Lys510
		π-Alkyl interaction	Tyr488
	7-1	Van der Waals	Phe61, Ala82
		Electrostatic	Gln64, Ala68, Asn74, Arg79, Met83, Glu86, Val331
		Carbon hydrogen bond interaction	Glu86
		Conventional hydrogen bond interaction	Asn74, Arg79
		Alkyl interaction	Ala82, Ala83
		π-Alkyl interaction	Phe61, Arg79, Ala82, Met83
		Amide-π stacked interaction	Ala82, Met83
		π-Sulfur interaction	Met83
	1USH	Van der Waals	Ala436, Met438, Lys441, Ala449, Pro507, Leu509, Lys512, Pro513, Gly514, Tyr515
		Electrostatic	Asp437, Glu442, Asp445, Tyr446
		Carbon hydrogen bond interaction	Asp437, Glu442, Pro513
		Alkyl interaction	Ala436, Met438, Leu509, Lys512
	11-5	Van der Waals	Tyr221, His226, Gly227, Ser228, Ser253, Asn511, Pro513, Gly514, Arg516
		Electrostatic	Asn229, His252, Gln254, Arg432, Asp510, Lys512, Lys515, Asn517, Gly519
		Carbon hydrogen bond interaction	Asn229, Asp510, Lys512, Lys515, Asn517
		Conventional hydrogen bond interaction	Arg432
		Halogen (fluorine) interaction	Asp510
		Alkyl interaction	Lys515
		π-Alkyl interaction	His252
	2DYT	Van der Waals	Ile129, Asp131, Ile132, Leu135, Leu243, Leu291, Phe293, Ser296, Thr295, His303, Thr306
		Electrostatic	Tyr209, Lys292
		Alkyl interaction	Ile132, Lys292, Leu243,
		π-Alkyl interaction	Lys292
		π-Sigma interaction	Ser296
	13-6	Van der Waals	Glu87, Gly157, Leu159, Pro299, Ile301, Gln302, Asp304
		Electrostatic	Asp77, Lys78, Val86, Gly88, Glu151, Glu152, Phe153, Ser300
		Carbon hydrogen bond interaction	Phe153, Pro299, Ser300, Ile301
		conventional hydrogen bond interaction	Glu152, His303
		π-interaction pair	His303
		π-Alkyl interaction	Phe153
		π-π Stacked interaction	His303

**Table 8:** The distance between the amino acid residue of the degrading enzyme and the target fluoroquinolone molecule before and after modification.

Degrading Enzyme	Amino Acid Residue	Distance from the Target Fluoroquinolone Molecule					
		Distance from CIP (Å)	Mean Distance (Å)	Distance from NOR (Å)	Mean Distance (Å)	Distance from OFL (Å)	Mean Distance (Å)
**1YZP**	Ile91	7.50	8.10	7.80	8.57	8.10	7.03
	Phe93	9.10		9.70		7.40	
	Ile100	7.70		8.20		5.60	
3-6	Arg91	18.50	19.80	23.40	24.17	22.70	24.20
	Arg93	20.20		24.70		25.00	
	Arg100	20.70		24.40		24.90	
**4DTE**	Phe181	6.20	5.50	9.10	6.77	5.30	4.57
	Val184	4.10		2.90		3.20	
	Pro349	6.20		8.30		5.20	
5-2	Cys181	15.70	15.33	18.40	16.83	23.30	23.80
	Ser184	17.50		18.40		26.90	
	Lys349	12.80		13.70		21.20	
**3NQA**	Val155	8.50	5.60	5.40	5.87	7.00	4.97
	Ile200	3.70		3.90		2.90	
	Val201	4.60		8.30		5.00	
6-1	Lys155	2.60	5.67	7.90	8.20	8.50	8.00
	Lys200	4.70		8.70		8.30	
	Lys201	9.70		8.00		7.20	
**1E1D**	Trp292	2.10	5.93	2.40	6.00	3.00	6.57
	Trp293	7.50		7.80		8.50	
	Tyr493	8.20		7.80		8.20	
7-1	Lys292	16.70	20.27	15.30	17.97	19.20	23.00
	Lys293	23.30		20.10		25.70	
	Lys493	20.80		18.50		24.10	
**1USH**	Val432	6.90	7.17	8.30	6.90	4.20	6.77
	Tyr515	8.10		6.60		8.20	
	Val516	6.50		5.80		7.90	
11-5	Arg432	11.00	7.60	6.20	6.40	10.10	7.63
	Lys515	5.50		6.30		6.90	
	Arg516	6.30		6.70		5.90	
**2DYT**	Ile136	8.20	8.50	7.80	8.13	7.80	7.90
	Met139	9.20		9.70		8.10	
	Leu258	8.10		6.90		7.80	
13-6	Lys136	17.70	19.77	15.80	17.37	11.20	13.27
	Arg139	13.70		11.80		10.60	
	Arg258	27.90		24.50		18.00	
